# Using formalin fixed paraffin embedded tissue to characterize the microbiota in p16-positive and p16-negative tongue squamous cell carcinoma: a pilot study

**DOI:** 10.1186/s12903-024-04051-w

**Published:** 2024-02-28

**Authors:** Zhan Yuanbo, Liu Tianyi, Song Xuejing, Liu Xinpeng, Wang Jianqun, Xu Wenxia, Geng Jingshu

**Affiliations:** 1 Department of Periodontology and Oral Mucosa, The Second Affiliated Hospital of Harbin Medical University, Harbin, China; 2 Department of pathology, Harbin Medical University Cancer Hospital, Harbin Medical University, Heilongjiang, Harbin, China; 3 Institute of Hard Tissue Development and Regeneration, The Second Affiliated Hospital of Harbin Medical University, Harbin, China; 4 Department of Pathology, the Second Affiliated Hospital of Harbin Medical University, Harbin, China; 5 Harbin Institute of Technology Hospital, Harbin, China

**Keywords:** Oral squamous cell carcinoma, 16S rRNA sequencing, Oral microbiota, Formalin-fixed paraffin-embedded tissue

## Abstract

**Background:**

Tongue squamous cell carcinoma (TSCC) is the most common oral cavity cancer, and p16 immunohistochemistry is an exact and available tool in the prognostic and predictive characterization of squamous cell cancers in the head and neck. Microorganisms have a close relationship with the development of TSCC. However, the association between oral bacteria and p16 status has not been well defined in the case of TSCC. Compared with traditional clinical microbial collection methods, formalin-fixed paraffin-embedded (FFPE) tissue samples have several advantages.

**Methods:**

To compare the microbiota compositions between p16-positive and p16-negative patients with TSCC, we performed a small pilot study of microbiological studies of TSCC by paraffin tissue. DNA from FFPE tissue blocks were extracted and microbiomes were profiled by sequencing the 16 S-rRNA-encoding gene (V1–V2/V3-V4/V4 regions). Alterations in the functional potential of the microbiome were predicted using PICRUSt, Tax4Fun, and BugBase.

**Results:**

A total of 60 patients with TSCC were enrolled in the study, however, some challenges associated with DNA damage in FFPE tissues existed, and only 27 (15 p16-positive and 12 p16-negative) passed DNA quality control. Nevertheless, we have tentatively found some meaningful results. The p16 status is associated with microbiota diversity, which is significantly increased in p16-positive patients compared with p16-negative patients. *Desulfobacteria, Limnochordia*, *Phycisphaerae, Anaerolineae*, *Saccharimonadia* and *Kapabacteria* had higher abundances among participants with p16-positive. Moreover, functional prediction revealed that the increase of these bacteria may enhance viral carcinogenesis in p16-positive TSCC.

**Conclusions:**

Bacterial profiles showed a significant difference between p16-positive TSCC and p16-negative TSCC. These findings may provide insights into the relationship between p16 status and the microbial taxa in TSCC, and these bacteria may provide new clues for developing therapeutic targets for TSCC.

**Supplementary Information:**

The online version contains supplementary material available at 10.1186/s12903-024-04051-w.

## Highlights

λ Some challenges associated with DNA damage in FFPE tissues indeed exist.

λ Microbiota diversity increased in p16-positive tongue squamous cell carcinoma(TSCC).

λ *Desulfobacteria* and other five bacteria have higher abundance in p16-positive TSCC.

λ The increase of these bacteria may enhance viral carcinogenesis in p16-positive TSCC.

**Supplementary Information:**

The online version contains supplementary material available at 10.1186/s12903-024-04051-w.

## Introduction

Oral cancer represents a major public health problem worldwide, and oral squamous cell carcinomas (OSCC) account for more than 90% of oral cancers [1]. Of these 90%, OSCCs of the tongue (TSCC)are reported to occur with rates of up to 40–50% [2]. TSCC is significantly more aggressive than other types of OSCC [3]. The etiology of cancer is complicated by the fact that a high proportion is attributable to environmental and heritable risk factors. An emerging concept in cancer biology implicates the microbiome as an influential environmental factor modulating the carcinogenic process [4]. Changes in the human microbiome are hypothesized to increase tumor formation and contribute to carcinogenesis through a few potential mechanisms: triggering cellular antiapoptotic signals; releasing carcinogenic factors; unleashing chronic inflammatory responses, and modifying anticancer immunity [5, 6].

The microbiota is a collection of microbial taxa associated with humans [7]. This microorganism is a double-edged sword that may have beneficial or deleterious effects on its host [8]. The benefit side: active communication (‘cross-talk’) between some resident bacteria and host cells has been proven, and micro-organisms are maintained in an environment that is supplied with a diverse array of host molecules that serve as nutrients [9]. The resident oral microbiota can also contribute to the host defenses by preventing the establishment of many exogenous micro-organisms [9]. More importantly, the normal oral microbiome is also vital in maintaining oral as well as systemic health [10]. The bad side: microorganisms and their products, including endotoxins (lipopolysaccharides), enzymes, and metabolic byproducts are toxic to host cells and may directly induce mutations or alter signaling pathways that affect cell proliferation and/or survival of epithelial cells, and eventually lead to cancer [11].

The occurrence and development of OSCC is a complicated process, and cell cycle regulation is crucial for tumorigenesis. Protein p16 is a cyclin-dependent kinase inhibitor that inhibits retinoblastoma protein (pRb) phosphorylation and blocks cell cycle progression at the G1 to S checkpoint [12]. The loss of p16 expression results in a worse prognosis for head and neck squamous cell carcinomas (HNSCC). Bova et al. [13] considered that the loss of p16 expression is associated with a reduction in the 5-year overall survival rate, and they also observed that overexpression of cyclin D1 and loss of expression of p16 are independent death predictors in TSCC [13]. In patients with TSCC, p16 positivity correlated with improved relapse-free survival [14]. Researchers also suggested that p16-positive HNSCC had better treatment outcomes than p16-negative HNSCC [15]. In addition, the expression of p16 occurs as a result of the functional inactivation of the pRb by the HPV E7 protein [16]. Some studies found that the p16 expression has high concordance with other methods of HPV-DNA detection, suggesting that p16 is a surrogate marker for HPV [17]. In another aspect, the role of oral microbiota on OSCC/TSCC has been raised. Researchers have proposed the hypothesis that oral bacterial infections can promote OSCC/TSCC carcinogenic processes [18, 19]. However, very little is currently known about the relationship between p16 status and the microbial taxa in TSCC. In previous studies on the microorganism of OSCC, including TSCC, the vast majority of survey data were detected from oral saliva [20–22] or fresh tissue samples [19]. However, compared with these traditional clinical microbial collection methods, pathological formalin-fixed paraffin-embedded (FFPE) tissue samples have the advantages of long-term storage at room temperature and retrospective analysis at any time. Moreover, FFPE tissues are widely available from biobanks in pathology departments. Paraffin samples can also be well used to conveniently detect the expression of specific proteins in disease states, such as p16 expression analysis by immunohistochemistry. Some researchers have studied the changes of microorganisms in tumors [11, 19] or other diseases (e.g., IgA nephropathy [23]) by paraffin tissue.

In this study, to investigate the difference in bacterial communities between p16-positive and p16-negative TSCC, we detected the microbiota changes by using the tongue paraffin tissue.

## Methods

### Sample collection

The study group comprised patients who were histopathologically diagnosed with TSCC (oral squamous cell carcinomas located exclusively in the tongue was included) at the Second Affiliated Hospital of Harbin Medical University and the Stomatological Hospital of China Medical University between 2017 and 2021 and had available FFPE tissue for analysis. All cases of TSCC from sites other than the tongue, potentially malignant disorders and any pseudo malignancies of head and neck region (ameloblastoma, angiolymphoid hyperplasia with eosinophilia, calcifying epithelial odontogenic tumor, inflammatory myofibroblastic tumor, necrotizing sialometaplasia, nodular fasciitis, pseudoepitheliomatous hyperplasia, respiratory epithelial adenomatoid hamartoma, spindle cell/pleomorphic lipoma, squamous odontogenic tumor) were excluded from the study. Cases submitted to radio/chemotherapy prior to surgical treatment or with any severe systemic disease (such as immune deficiency or autoimmune) were excluded.

All paraffin specimens used in this study were obtained from the archives of pathology laboratories and those paraffin specimens were the remaining samples after routine pathological examination. Two experienced oral pathologists rectified the presence of tumor cells in the collected tissues by examination of histological sections stained with hematoxylin and eosin (HE). Clinical data such as age, gender, differentiation degree (DD), tumor lymphatic metastasis (TM), and muscle infiltration (MI) were extracted from the histopathological reports and medical records (Additional file 1: Supplementary Table S1). The necessary ethical approval for our study was approved by the Institutional Review Board of Second Affiliated Hospital of Harbin Medical University (Approval NO.KY2022-179). Waiver of informed consent was approved by the Institutional Review Board of Second Affiliated Hospital of Harbin Medical University (NO.KY2022-179). All methods were carried out in accordance with the relevant regulations.

### Hematoxylin and Eosin (H&E) staining and immunohistochemistry (IHC)

Serial sections were prepared at a thickness of 4-µm for hematoxylin and eosin (H&E) staining. According to conventional World Health Organization (WHO) grading [24], cases were categorized into 3 grades i.e. well differentiated TSCC, moderately differentiated TSCC and poorly differentiated TSCC. The grading was determined by analyzing the degree of keratinization, cellular differentiation, nuclear changes, lymphoplasmablastic infiltration, and the invasive front. Immunohistochemistry was performed on formalin-fixed and paraffin-embedded 4-µm-thick sections. Tissue sections were deparaffinized in xylene and rehydrated in a decreasing ethanol series. Sodium citrate heat-induced (about 120˚C) antigen retrieval was performed by a pressure cooker. Endogenous peroxidases were blocked with peroxide block (GTVisionTM + Detection system/Mo&Rb, medium A) for 15 min at room temperature and washed in distilled water. The sections were incubated with primary anti-p16 antibody (Gene Tech, #GT233004) overnight incubation at 4˚C, and incubated at room temperature for 1 h with biotinylated secondary antibodies (GTVisionTM + Detection system/Mo&Rb, medium B) for 30 min at room temperature followed by washing with Phosphate Buffer Saline (PBS). Color was developed using the chromogen diaminobenzidine (DAB) for 5 min, and the sections were washed in buffer followed by water and counterstained with Harris hematoxylin, air dried, and mounted with neutralresinsize. The negative control group in which the primary antibody was replaced with PBS did not demonstrate any positive reactions.

### DNA extraction and quality control

For each sample, the first few scrolls of the FFPE blocks were discarded, and then 10 μm*5 scrolls were cut down into sterile 2 ml centrifuge tubes. TSCC FFPE tissue microbial total DNA was extracted using the FastDNA Spin Kit for soil. DNA was quantified by TBS-380 fluorometer (Turner BioSystems Inc., Sunnyvale, CA). High quality DNA (OD260/280 = 1.8 ~ 2.0, > 20ug) was used for further research. A NanoDrop 2,000 UV VIS spectrophotometer (Thermo Scientific, Wilmington, USA) was used to test the concentration and purification of the final DNA, and 2% agarose gel electrophoresis showed the DNA quality. DNA quality is a critical step for subsequent microbial sequencing detection; therefore, we controlled DNA quality by two polymerase chain reactions (PCRs) and PCR products were detected by agarose gel electrophoresis. The results were divided into three grades according to the band and concentration of the PCR product: A, the target band of the PCR product was correct in size and concentration, which could be used for subsequent experiments; B, the size of the target band of the PCR product was correct, but the concentration was low, so the subsequent experiments could be attempted; and C, the target band of the PCR product was too weak or undetectable for subsequent experiments.

### 16 S rRNA gene sequencing

DNA was amplified by PCR with primers (27 F/338R, 338 F/806R and 515 F/806R, Table 1) targeting the hypervariable V1–V2 region, the V3–V4 region and the V4 region of the 16 S rRNA gene. Parallel tagged sequencing was performed to sequencing using an Illumina MiSeq sequencer (Illumina, San Diego, CA, USA). The following criteria were used for sequence assembly and filtering: 1) filtering the base of reads tail with a mass value below 20, and setting a 50 bp window. If the average mass value in the window was lower than 20, the back-end base was cut off from the window, and the N-base reads were removed;2) According to the overlap between PE reads, pairs of reads were merged into a sequence, with a minimum overlap length of 10 bp; 3) The maximum mismatch ratio allowed for overlap of spliced sequences was 0.2, and the unqualified sequences were screened.4) The samples were differentiated according to the barcode and primer at both ends of the sequence, and the direction of the sequence was adjusted. The allowable mismatch number of the barcodes was 0, and the maximum mismatch number of the primer was 2. To facilitate the analysis, we obtained high-quality sequences with a similarity of 97% clustering operational taxonomic units (OTUs) using the USEARCH (v7.0; http://drive5.com/uparse/), and UCHIME was utilized to identify and remove chimeric sequences.

**Table 1:** Primers used for 16 S rRNA gene sequencing analysis

Region	Name	F/R	Sequence
V1–V2	27 F/338R [25]	F	5′-TCGTCGGCAGCGTCAGATGTGTATAAGAGACAG-3′
		R	5′-GTCTCGTGGGCTCGGAGATGTGTATAAGAGACAG-3′
V3-V4	338 F/806R [26]	F	5′-ACTCCTACGGGAGGCAGCAG-3′
		R	5′-GGACTACHVGGGTWTCTAAT-3′
V4	515 F/806R [23]	F	5′‑GTGCCAGCMGCCGCGG-3′
		R	5′GGACTACHVGGGTWTCTAAT-3′

### Bioinformatics and statistical analysis

The data generated from Illumina platform were used for bioinformatics analysis. All of the analyses were performed using I-Sanger Cloud Platform (www.i-sanger.com) from Shanghai Majorbio. The detailed procedures were as follows, alpha diversity analysis and rarefaction analysis were performed using the Mothur v 1.30.2 and plot-rarefaction (Majorbio). Unweighted Principal Component Analysis (PCA) at the OTU level and the Partial Least Squares Discriminant Analysis (PLS-DA) at the genus level were used to evaluate the beta diversity. Differences in the abundance of microbiota between p16-positive and p16-negative samples were determined by Wilcoxon test followed by multiple comparisons using FDR *p*-value correction, and *P* < 0:05 was considered statistically significant. The Spearman rank test was applied to analyze the correlation between the bacterial composition (family level) and the clinical characteristics of the tongue cancer patients, including age, gender, differentiation degree (DD), tumor metastasis (TM), and muscle infiltration (MI).

### Gene prediction and annotation

The functional potentials of the microbiome were inferred from the taxonomic compositions determined by the 16 S rRNA gene survey using PICRUSt [27], Tax4Fun [28], and BugBase [27]; The OTU abundance was standardized by PICRUSt and then the Kyoto Encyclopedia of Genes and Genomes (KEGG) Ortholog (KO) information corresponding to OTU were obtained by the greengene ID corresponding to each OTU. In Tax4Fun, 16 S RNA gene sequences were functionally annotated by converting the 16 S taxonomic lineages based on Silva database into the taxonomic lineages of prokaryotes in KEGG database. By BugBase, the OTU was normalized with the predicted 16 S copy number and then predicted microbial phenotypes using the pre-calculated files provided, and the phenotypes included gram positive, gram negative, biofilm forming, pathogenic and mobile element containing, oxygen utilization and oxidative stress tolerance.

## Results

### Sample collection and DNA quality control

**Fig. 1:**
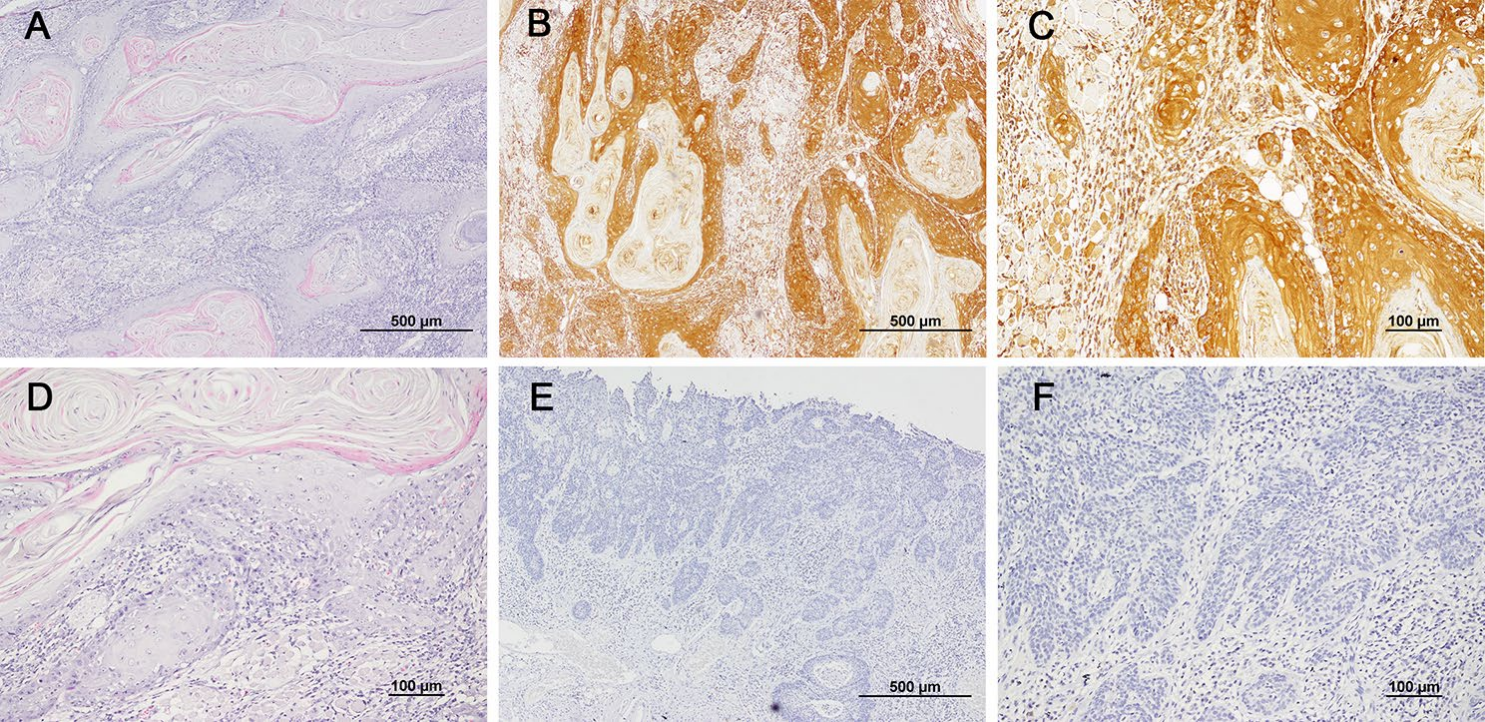
H&E Staining and p16 Immunohistochemistry (IHC) in TSCC. **A,D**:the 40X and 100x of H&E staining; **B,C**: 40Xand 100X of the p16 positive; **E,F**: 40Xand 100X of the p16 negative

In total, 60 paraffin tissue samples of TSCC were collected from the Stomatological Hospital of China Medical University (32 patients) and the Second Affiliated Hospital of Harbin Medical University (28 patients). In our study, the DNA quality is shown in Table 2. In general, the success rate of samples meeting the needs of further library construction and sequencing is not very high. The quality of DNA detected by different PCR primers was not completely consistent. The primer 338 F/806R had the lowest success rate (8.33%), and the primer 27 F/338R had the highest success rate (18.33%). Paraffin samples from different hospitals and different years did not change the success rate of amplification(*P* > 0.05, Additional file 1: Supplementary Table S2-3). It is worth noting that we re-cut 32 identical samples of paraffin volume for a second DNA quality assessment, but could still not improve the success rate of library construction. In addition, the number of samples that met the criteria for primer 338 F/806R was too small, hence subsequent analyses did not include this primer. Among the 12 samples that met the requirements for library construction by 515 F/806R, 6 samples were p16 positive and 6 samples were p16 negative. Among the 15 samples that met the requirements by 27 F/338R, 9 samples were p16 positive and 6 samples were p16 negative. The p16 immunohistochemistry is shown in Fig. 1. There was no significant difference between the p16 positive and negative for gender and age.

**Table 2:** DNA quality assessment by different primers

Primer	The number of samples	PCR pre-experiment				PCR formal experiments				Success rate of sequencing library construction
		A	B	C		A	B	C		A + B/ total number of cases
515 F/806R	60	0	12(6 p16+/6 p16-)	48		8	2	2		16.67%
27 F/338R	60	0	15(9 p16+/6 p16-)	45		10	1	4		18.33%
338 F/806R	60	0	5(3 p16+/2 p16-)	55		5	0	0		8.33%

### Alpha diversity of the microbiome between the p16-positive group and p16-negative group

#### Sequence by primers 515 F/806R

**Fig. 2:**
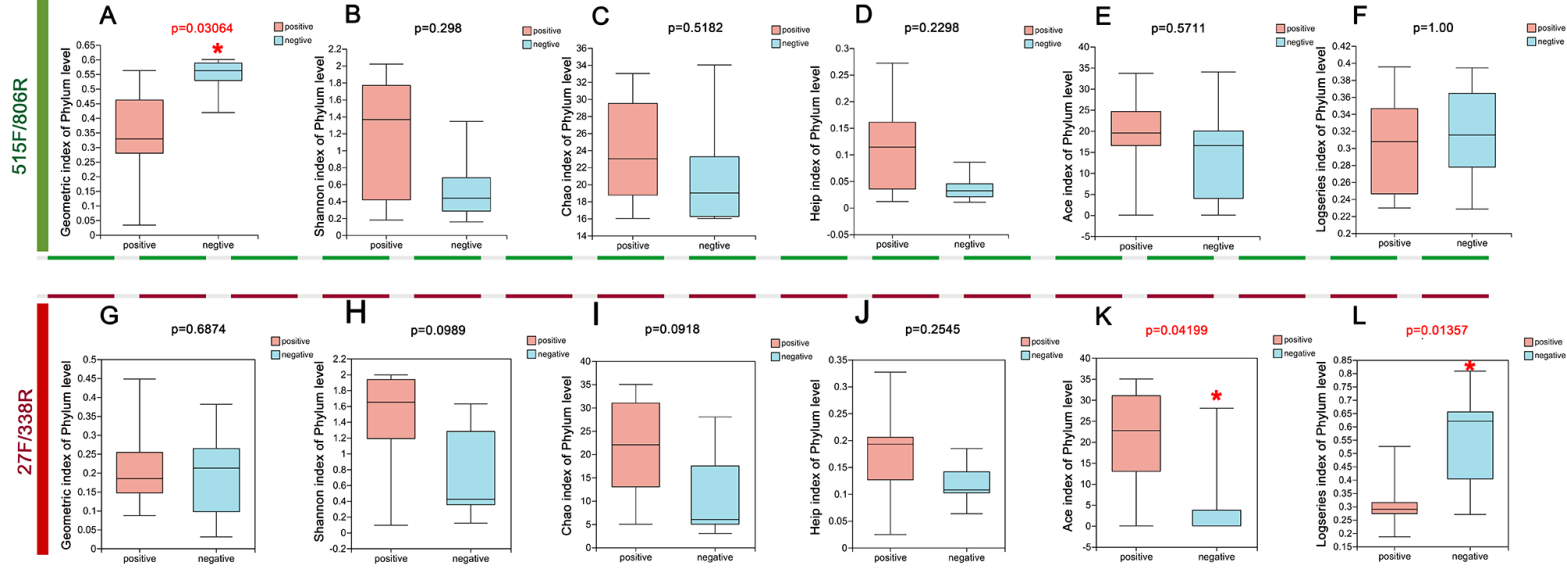
The diversity estimator of p16-positive patients and p16-negative patients on the level of Phylum. **A-F**, community richness estimator (Geometric, Shannon, Chao, Heip, Ace and logseries index) from the prism 515 F/806R; **G-F**, community richness estimator (Geometric, Shannon, Chao, Heip, Ace and logseries index) from the prism 27 F/338R;

By detecting the bacterial reads of TSCC from all patients. After quality control, we obtained 633,581 reads, the number of bases was 162,175,858 bp and the sequence average length was 255 bp, and the number of genera was 948, of OTUs was 2345 from all the samples (Table 3). At the classification level of OTUs, there were no statistically significant differences in the community richness estimator (Sobs index; Chao index), the diversity estimators (Shannon index and logseries index) or the community evenness (Heip index) between p16 positive patients and p16 negative patients (Fig. 2B-F). However, at the phylum classification level, the diversity estimator (geometric index) in p16 positive patients was significantly lower than that in p16 negative patients (Fig. 2A).

#### Sequence by primers 27 F/338R

After quality control, we obtained 944,542 reads, the number of bases was 289,425,899 bp and the sequence average length was 306 bp, and the number of genera was 1144, and the number of OTUs was 4179 from all the samples (Table 3). At the classification level of Phylum, there were no statistically significant differences in the diversity estimator (geometric index, Fig. 2G) and the community richness estimator (Shannon index, Chao index, Heip index) between p16-positive patients and p16-negative patients (Fig. 2H-I, these results are similar to sequence by primers 515 F/806R). However, at the phylum level, the diversity estimator (ace index and logseries index) was significantly different between p16-positive patients and p16-negative patients (Fig. 2K, L).

**Table 3:** Diversity data analysis: species annotation result statistics

Primer	Reads	Bases(bp)	sequence average length(bp)	Phylum	Class	Order	Family	Genus	Species	OTUs
515 F/806R	633,581	162,175,858	255	49	132	299	481	948	1524	2345
27 F/338R	944,542	289,425,899	306	46	136	319	531	1132	2100	4103
338 F/806R	240,228	100,459,369	418	34	100	220	359	710	1206	1908

### Beta diversity of microbiome between p16-positive group and p16-negative group

#### Sequence by primers 515 F/806R

**Fig. 3:**
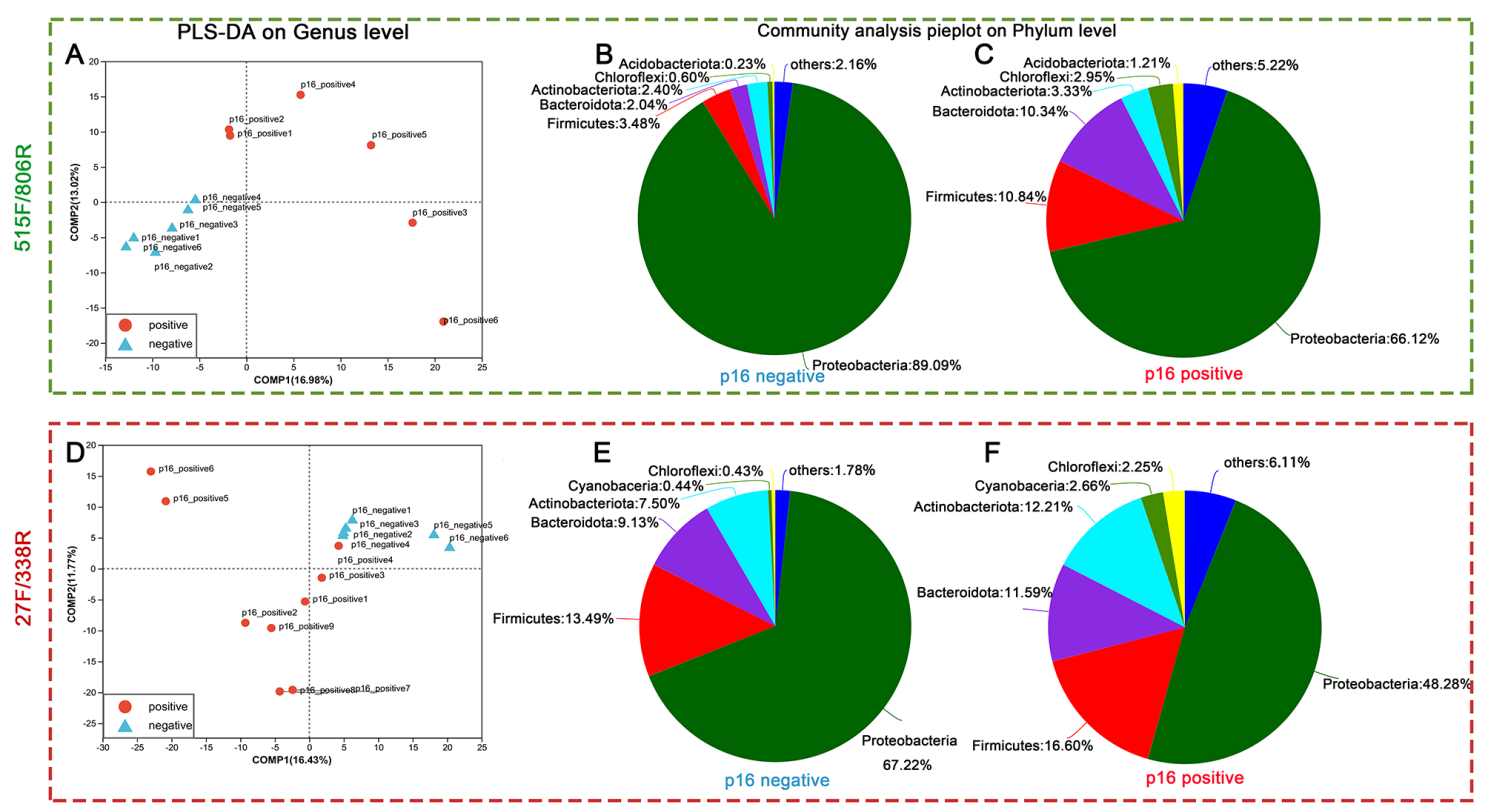
The Partial Least Squares Discriminant Analysis(genus level) and community analysis pieplot (Phylum level). The p16-positive group and the p16-negative group could be separated by the Partial Least Squares Discriminant Analysis (PLS-DA), **A**, PLS-DA of the p16-positive group and the p16-negative group from the prism 515 F/806R; **D**, PLS-DA of the p16-positive group and the p16-negative group from the prism 27 F/338R; **B, C** community analysis pieplot of p16-positive group and the p16-negative group (using prism 515 F/806R); **E,F**, community analysis pieplot of p16-positive group and the p16-negative group (using prism 27 F/338R)

The composition of microbiota in different samples was similar, but there were also different genera. The Unweighted Principal Component Analysis (PCA) at the OTU level was employed and indicated no obvious separation between groups. However, at the genus level, the p16-positive group and the p16-negative group could be separated by the Partial Least Squares Discriminant Analysis (PLS-DA) (Fig. 3A, D.) Interestingly, the p16-negative samples were clustered together, however, the samples of p16-positive were more discrete, which may imply that the microbiome varies greatly between samples within the p16-positive groups.

#### Sequence by primers 27 F/338R

The results from primers 27 F/338R were similar to those produced with primers 515 F/806R, which also showed that the composition of microbiota in different samples was similar, but there were also different genera. PCA at the OTU level indicated no obvious separation between groups. However, at the genus level, the p16-positive group and the p16-negative group could be separated by PLS-DA (Fig. 3D). Interestingly, the p16-negative samples were clustered together, however, the p16-positive samples were more discrete, which may also imply that the microbiome varies greatly between samples within the p16-positive groups.

### Common bacterial taxa between p16-positive group and p16-negative group

#### Sequence by primers 515 F/806R

The predominant bacterial components of samples from the p16-positive group and the p16-negative group were similar. *Proteobacteria*, *Firmicutes*, *Bacteroidota*, *Actinobacteriota* and *Chloroflexi* were the five most dominant phyla in the p16-positive group and p16-negative group (Fig. 3B,C). The proportion of the *Proteobacteria* in the p16-negative group (89.09%) was higher than that in the p16-positive group (66.12%). However, the proportion of the *Firmicutes* in the p16-positive group (10.83%) was higher than that in the p16-negative group (3.47%), and the proportion of the *Bacteroidota* in the p16-positive group (10.34%) was higher than that in the p16-negative group (2.04%). At the genus level, *Pseudomonas* and *Acinetobacter* were the two most dominant phyla in the p16-positive group and the p16-negative group.

#### Sequence by primers 27 F/338R

Similar to the sequence obtained by primers 515 F/806R, the predominant bacterial components of the samples between the two groups were similar. *Proteobacteria*, *Firmicutes*, *Bacteroidota* and *Actinobacteriota* were the four most dominant phyla in p16-positive group and p16-negative group (Fig. 3E,F). However, the fifth predominant bacterial component was *Cyanobacteria*, which was different from the results from the sequence by primers 515 F/806R(*Chloroflexi*); In addition, the percentage of dominant microbiota was also not the same. For instance, the proportion of the *Proteobacteria* in the p16-negative group (67.22%) was higher than that in the p16-positive group (48.28%). However, the proportion of the *Firmicutes* in the p16-positive group (16.60%) was higher than that in the p16-negative group (13.49%), and the proportion of the *Bacteroidota* in the p16-positive group (11.59%) was higher than that in the p16-negative group (9.13%). At the genus level, *Pseudomonas* and *Acinetobacter* were the two most dominant phyla in the p16-positive group and the p16-negative group.

### Distinct bacterial taxa between the p16-positive group and p16-negative group

#### Sequence by primers 515 F/806R

**Fig. 4:**
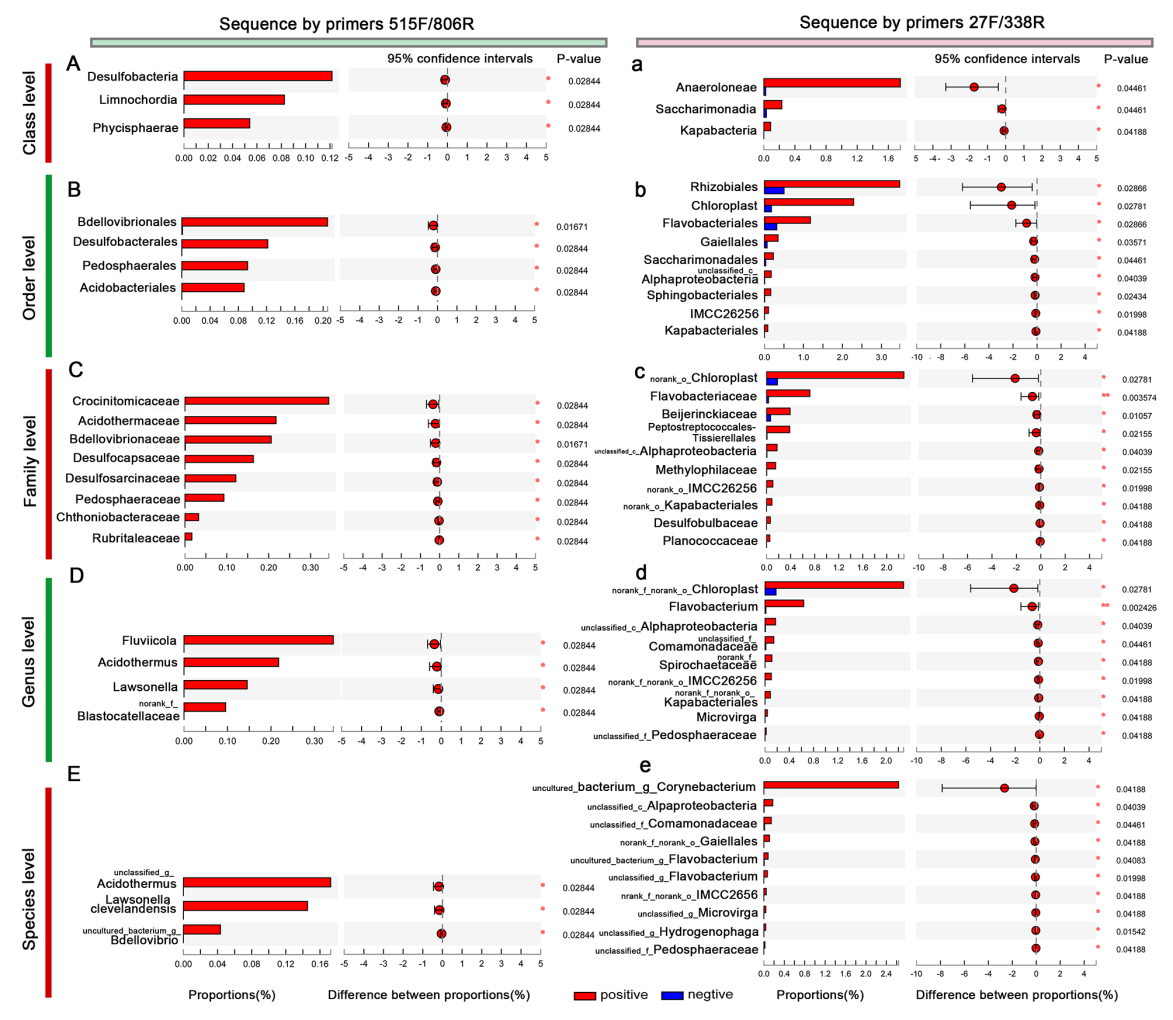
Distinct taxa identified in p16-positive group and p16-negative group

Some bacterial compositions of the p16-positive group samples varied from those of the p16-negative group. The microbiomes of the p16-positive samples and the p16-negative samples were analyzed at different taxonomic levels. At the class level, *Desulfobacteria Limnochordia*, and *Phycisphaerae* were found only in the p16-positive group (Fig. 4). At the order level, *Bdellovibrionales* exhibited significantly higher abundances in the p16-positive group than in the p16-negative group, and *Desulfobacterales, Pedosphaerales and Acidobacteriales* were found only in the p16-positive group. At the family level, 8 taxa exhibited significantly higher abundances in the p16-positive group than in the p16-negative group, including *Crocinitomicaceae, Acidothermaceae, Bdellovibrionaceae, Desulfocapsaceae, Desulfosarcinaceae, Pedosphaeraceae, Chthoniobacteraceae* and *Rubritaleaceae.* At the genus level, *Fluviicola, Acidothermus, Lawsonellanora*, and *nk_f__Blastocatellaceae* only existed in the p16-positive group.

#### Sequence by primers 27 F/338R

At the class level, the abundances of *Anaerolineae*, *Saccharimonadia* and *Kapabacteria* in the p16-positive group were significantly higher than those in p16-negative patients. At the order level, the abundances of *Rhizobiales, Chloroplast, Flavobacteriales, Gaiellales, Saccharimonadales, unclassified_c__Alphaproteobacteria, Sphingobacteriales, IMCC26256* and *Kapabacteriales* in the p16-positive group were significantly higher than those in p16-negative patients (Fig. 4). At the family level, *norank_o__Chloroplast, Flavobacteriaceae, Beijerinckiaceae, norank_o__Peptostreptococcales-Tissierellales, unclassified_c__Alphaproteobacteria, Methylophilaceae, norank_o__IMCC26256, norank_o__Kapabacteriales, Desulfobulbaceae* and *Planococcaceae* exhibited significantly higher abundances in the p16-positive group than that in the p16-negative group. At the genus level, *norank_f__norank_o__Chloroplast, Flavobacterium, unclassified_c__Alphaproteobacteria, unclassified_f__Comamonadaceae, norank_f__Spirochaetaceae, norank_f__norank_o__IMCC26256, norank_f__norank_o__Kapabacteriales, Microvirga*, and *unclassified_f__Pedosphaeraceae* exhibited significantly higher abundances in the p16-positive group than that in the p16-negative group. At the phylum level, *Patescibacteria* exhibited significantly higher abundances in the p16-positive group than that in the p16-negative group.

### Correlation analysis between the microbiomes and clinical characteristics

#### Sequence by primers 515 F/806R

**Fig. 5:**
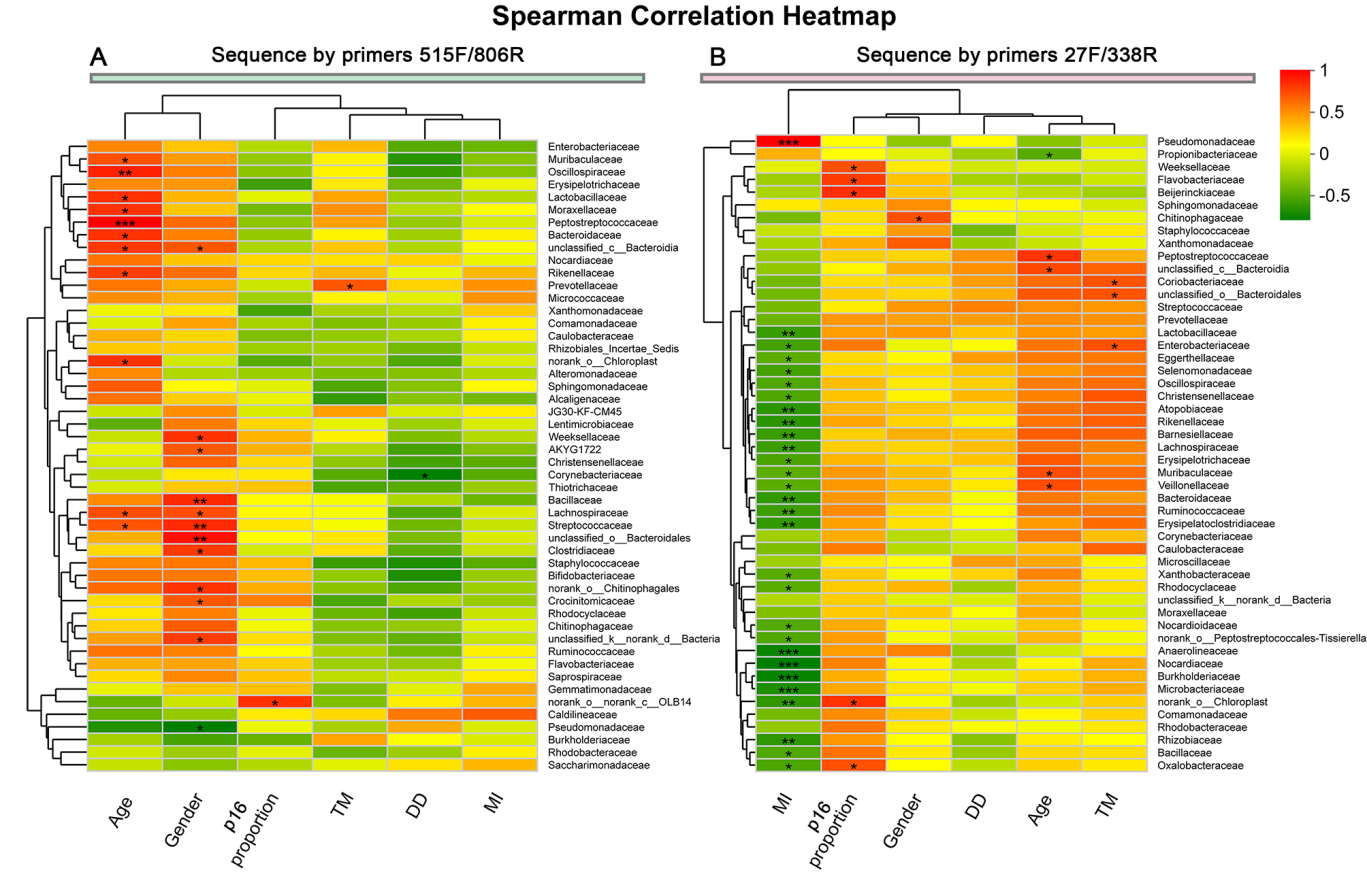
The correlation between the bacterial composition (family level) associated with the clinical characteristics. DD, differentiation degree; TM, tumor lymphatic metastasis; MI, muscle infiltration; *p <0.05;**p <0.01; ***p <0.001

The Spearman rank test was applied to analyze the correlation between the bacterial composition (family level) associated with the clinical characteristics. We considered possible interfering factors, including age, gender, and differentiation degree (DD), tumor lymphatic metastasis (TM), muscle infiltration (MI). Figure 5 displays partial Spearman correlation coefficients between 50 bacterial families and clinical parameters. We found that the abundances of *Peptostreptococcaceae*, *Oscillospiraceae*, norank_o__Chloroplast, *Bacteroidaceae*, *Lactobacillaceae, Moraxellaceae, unclassified_c__Bacteroidia, Rikenellaceae, Lachnospiraceae, Muribaculaceae, Streptococcaceae and Sphingomonadaceae were* positively correlated with the age. The abundance of *Corynebacteriaceae* was negatively correlated with DD (*r*= -0.61364, *p* = 0.03). The abundance of *Prevotellaceae* was positive for lymph node metastasis (*r* = 0.5864, *p* < 0.05).

#### Sequence by primers 27 F/338R

Similar to sequence by primers 515 F/806R, the correlations between the bacterial composition (family level) associated with the clinical characteristics were analyzed (Fig. 5). The abundances of Corynebacteriaceae and DD had a negative correlation trend(*r*=-0.17827), but there was no statistical significance (*p* = 0.525). The abundances of *Prevotellaceae* were positive for the lymph node metastasis (*r* = 0.3198, *p* = 0.24 > 0.05). The abundance of *Beijerinckiaceae*(*r* = 0.68781, *p* < 0.01), *norank_o__Chloroplast*(*r* = 0.67752,*p* = 0.01),*norank_o__Peptostreptococcales-Tissierellales*(*r* = 0.62898,*p* = 0.01201), *Anaerolineaceae*(*r* = 0.62611,*p* = 0.01) *Flavobacteriaceae*(*r* = 0.60772,*p* = 0.01), *Oxalobacteraceae*(*r* = 0.60011,*p* = 0.02) *and Rhizobiaceae* (*r* = 0.55293,*p* = 0.03) had positive correlation with the p16 proportion. One bacterium, *Pseudomonadaceae* was positive for MI (*r* = 0.78743, *p* < 0.05).

### Microbial functions altered in the p16-positive group and p16-negative group

#### Sequence by primers 515 F/806R

**Fig. 6:**
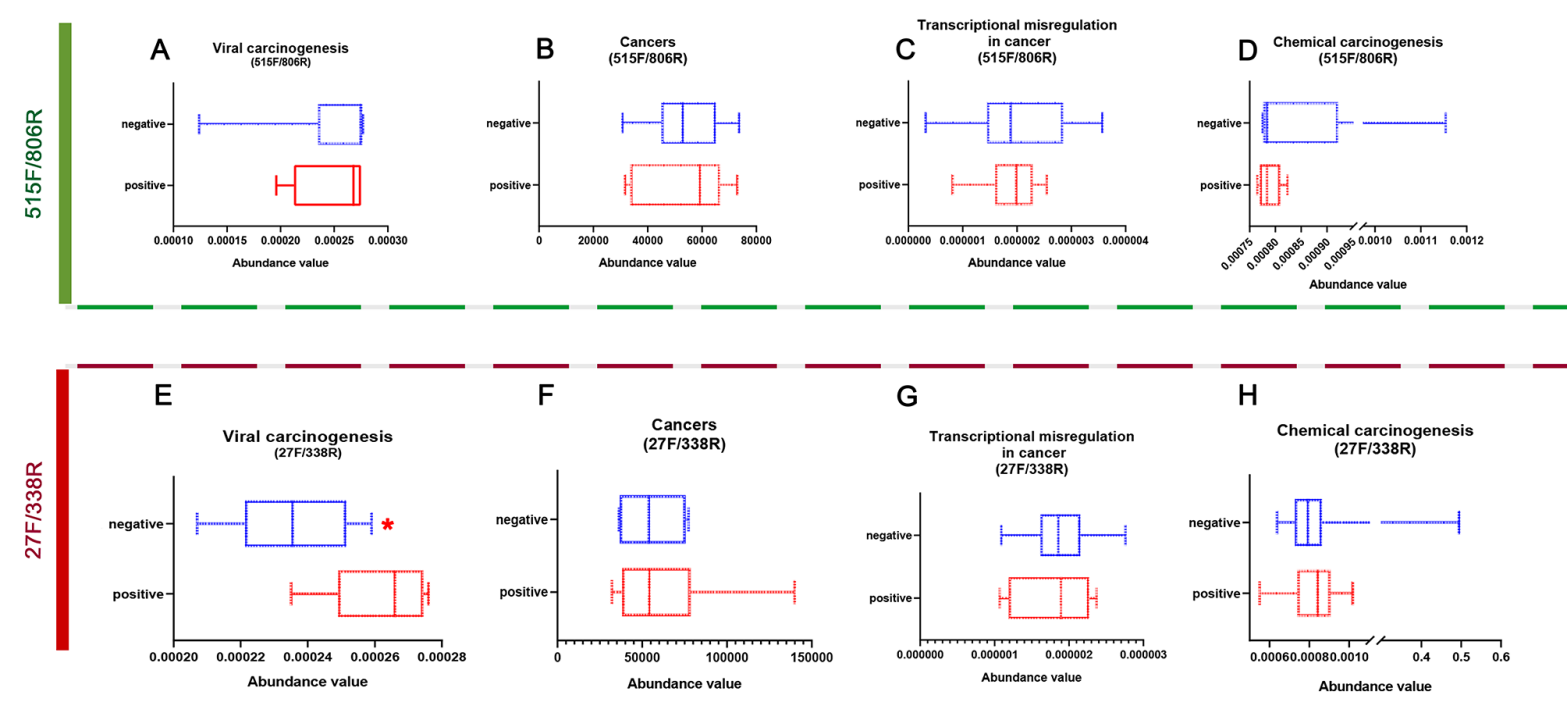
Pathways associated with human diseases(cancer) were predicted by using the Tax4Fun function. **A,E**, viral carcinogenesis; **B,F**, Cancers; **C,G**, Transcriptional misregulation in cancer; D,H, Chemical carcinogenesis. **A-D**, results from the prism 515 F/806R; E-F,results from the prism 27 F/338R.

To characterize the functional alterations of the microbiome in the p16-positive group and the p16-negative group, we predicted the functional composition profiles from 16 S rRNA sequencing data with PICRUSt, Tax4Fun and BugBase in the p16-positive group and the p16-negative group. Statistically significant KEGG pathways for each group were determined. By using the PICRUSt, compared to the p16-negative group, the p16-positive group was significantly enriched for pathways involved in germination, flavone and flavonol biosynthesis, cell cycle, mTOR signaling pathway, and hepatitis C (Additional file 2: Supplementary Table S4). By using the Tax4Fun function prediction, the human diseasesC pathways associated with cancer were both enriched for the p16-negative group and the p16-positive group, including Pathways in cancer, Chemical carcinogenesis, Transcriptional misregulation in cancer, Viral carcinogenesis (Fig. 6 and Additional file 2: Supplementary Table S5). At the organism level, functions related to aerobic, mobile element containing, biofilm forming, Gram_Negative and Potentially_Pathogenic were depleted in the p16-positive group. However, functions related to Facultatively-Anaerobic and Gram_Positive were slightly enhanced in the p16-positive group, although there was no significant difference.

#### Sequence by primers 27 F/338R

**Fig. 7:**
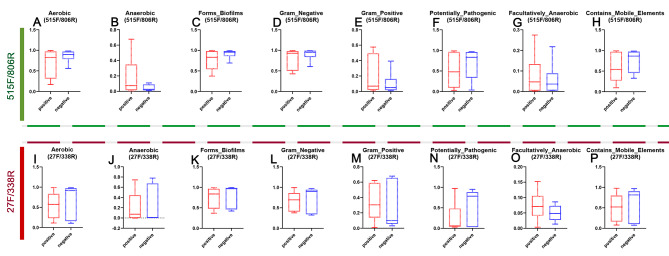
Phenotype prediction by using the BugBase. **A,I**, aerobic; **B,J**, Anaerobic; **C,K**, biofilm forming; **D,L**, Gram_Negative; **E,M**, Gram_Positive; **F,N**, Potentially_Pathogenic, **G,O**, Facultatively-Anaerobic; **H, P**, mobile element containing; **A-D**, results from the prism 515 F/806R; **E-F**, results from the prism 27 F/338R.

Unlike the results from the sequence by primers 515 F/806R, we found that the enriched pathways between the p16-negative group and the p16-positive group had no significant difference by using the PICRUSt (Additional file 3: Supplementary Table S6). However, the filtered pathways associated with human diseases(cancer) were similar to those from primers 515 F/806R by using the Tax4Fun function prediction (Fig. 6 and Additional file 3: Supplementary Table S7). Specifically, four pathways were significantly different between the p16-negative group and the p16-positive group, including viral carcinogenesis (*P* = 0.01, Fig. 6). At the organism level, it was similar to that from primers 515 F/806R: functions related to aerobic, mobile element containing, biofilm forming, Gram_Negative and Potentially_Pathogenic were depleted in the p16-positive group. However, Anaerobic, functions related to Facultatively-Anaerobic and Gram-Positive were slightly enhanced in the p16-positive group, although there was no significant difference (Fig. 7). These data show a shift toward more anaerobic and gram-positive bacteria in specimens with p16-positive.

## Discussion

Generally, OSCC is caused by the long-term impact of known risk factors: tobacco and alcohol, along with chronic traumatization. In recent years, researchers have also paid more attention to the role of bacteria in many cancers, from gastric cancer (*Helicobacter pylori* [29]), which was first discovered, to gallbladder cancer (*Salmonella typhi* [30]). Oral carcinogenesis is also associated with bacteria [31–33]. P16 is a cellular protein involved in cell cycle regulation and is expressed at a very low level in normal cells. The expression of p16 is associated with oral tumor stage and progression [34], making it helpful for determining treatment prognosis in OSCC [15]. Recently, meta-analysis suggests that p16 overexpression in oral potentially malignant disorders is significantly associated with a greater risk of malignant transformation to OSCC [35]. However, in the case of OSCC, the relationship between p16 status and oral bacteria has not been thoroughly characterized. Therefore, a detailed analysis of the microbial status in p16-positive or p16-negative OSCC is necessary.

In the human mouth, most microbes are site specialists, the most important cause of this is that the mouth is not a unitary environment and sites within the mouth, although connected by salivary flow, constitute distinct habitats [36]. Hence, to avoid the influence of different sites on the results, only tongue squamous cell carcinoma was selected in this study.

With the emergence of next-generation sequencing (NGS), 16 S rRNA sequencing promoted the study of associations between microbiota and OSCC. Several studies [11, 33, 37–39] on oral microorganisms and OSCC have been carried out using this method. Among these studies, different samples were collected, including oral rinse [38], saliva [11] and fresh tumor samples [33, 37, 39]. Compared with these traditional clinical microbial collection methods, FFPE tissue samples have several advantages, such as long-term storage at room temperature, and retrospective analysis at any time, representing a vast repertoire of genetic information and are widely available from biobanks in pathology departments. However, some challenges associated with FFPE tissues may also exist. For instance, for formalin-treated samples, retrieving nucleic acids from these tissues for downstream molecular analysis has proven challenging for researchers [40]. Formalin fixation mainly induces damage in DNA by creating cross-links (DNA-DNA, protein-DNA), depurination, DNA fragmentation and sequence alterations.

In the present study, we encountered similar challenges. DNA extraction is one of the first challenges. Our team’s previous study detected the tonsillar microbiota changes in formalin-fixed paraffin-embedded tissue by the QIAamp DNA FFPE tissue kit [23], however, the DNA extraction was not particularly satisfying. Hence, according to the previous experience of our teams, the advice from the sequencing companies and the literature report (DNA yields were significantly higher with either method of the FastDNA kit for soil than with the other kit, such as MoBio kit, Mobio Ultra Clean® Fecal DNA Isolation Kit; QIAamp® DNA Stool Mini Kit and FastDNA® SPIN Kit [41, 42], we ultimately used the Fast DNA Spin Kit for soil to extract the DNA. It is crucial to note that we do not necessarily mean that this kit is the best, rather, we indicate that the DNA extraction procedure still needs to be improved. Another challenge is choosing the appropriate primers. We found that the replacement of some primers could not effectively improve the success rate of amplification. Some primers even reduce the success rate of amplification. This suggests that it is very important for us to choose appropriate primers in future studies. Moreover, storage time has also been considered a limiting factor for retrieving intact nucleic acids from FFPE samples [43]. However, in our study, although there was no significant difference in the amplification efficiency of paraffin samples for different storage times, we noticed that the success rate of DNA amplification of paraffin samples with a short storage time had a higher trend than that of paraffin samples with a long storage time. Therefore, when selecting paraffin samples for analysis, the storage time of paraffin samples should be considered as much as possible to ensure the repeatability and reliability of the results. At the technical level, previous researchers [44] detected the bacteria in blood samples from healthy and diseased humans, in which a nested-PCR was applied for DNA amplification and 16 S library preparation. Normally, bacterial DNA is present at very low abundance in the blood of healthy samples. In these situations, nested-PCR is necessary to increase the sensitivity and/or specificity of PCR [45]. Hence, in future studies, the nested-PCR method may be an alternative method for DNA amplification in paraffin samples with low DNA content.

Overall, the results of 515 F/806R and 27 F/338R are quite similar, for example, the four most dominant phyla in the p16-positive group and p16-negative group are identical, including *Proteobacteria*, *Firmicutes*, *Bacteroidota*, and *Actinobacteriota.* These most dominant phyla in the oral cavity match those observed in earlier studies [39, 46]. However, the results of 515 F/806R and 27 F/338R had some subtle differences, such as the diversity estimator of p16-positive patients and p16-negative patients at the phylum level being not the same. The possible reasons are mainly due to the sequencing length and capturing species of bacteria by different primers not being entirely consistent.

The current study identified a few bacterial classes with higher abundance among participants with p16 positivity, including *Desulfobacteria, Limnochordia*, *Phycisphaerae, Anaerolineae*, *Saccharimonadia* and *Kapabacteria. Desulfobacteria* is one of the six classes of dissimilatory phosphite oxidation microorganisms and can be detected in the environment. The role of this microbiome in human disease has not been fully reported. A recent report [47] using concatenated protein marker trees resolved the class *Thermodesulfobacteria*(*Desulfobacteria*) as a clade within the *Deltaproteobacteria*, which have the potential to play a role in the etiology of periodontal disease [48]. Periodontitis can be considered as an independent risk factor for oral cancer [49]. It is hypothesized that hydrogen sulfide produced by *Desulfobacteria* as a virulence factor may damage the intestinal epithelium [50] or periodontal tissue [51] leading to cell death and chronic inflammation. Therefore, the role of this genus in oral cancer requires further investigation. *Limnochordia* is placed in the phylum *Firmicutes* [52]. In the present study, the proportion of the *Firmicutes* in the p16-positive group was higher than that in the of p16-negative group. To date, no evidence has been found associating *Limnochordia* with oral cancer. Regarding *Phycisphaerae*, although its role in human cancer has not yet been reported, a bacterial community analysis showed that reduced enrichment of *Phycisphaerae* in the intestinal genera was observed in decreasing tendency from nonsmokers without hypertension, smokers without hypertension, and nonsmokers with hypertension to smokers with hypertension [53]. This highlights the impact of smoke on the intestinal genera, especially *Phycisphaerae.* Smoke is one of the main risk factors for TSCC, and future studies regarding the role of *Phycisphaerae* in TSCC are therefore recommended. *Saccharimonadia*, formerly known as phylum TM7, are ubiquitous members of the human oral microbiome, accumulating evidence linking their association with periodontal disease [54]. Moreover, TM7 could modulate the oral microbiome structure hierarchy and functionality by affecting their bacterial host physiology [54]. However, there are still many unanswered questions about whether this microbiota could affect tumor development. For the *Anaerolineae*, it was positively correlated with the levels of the cytokines, IL-2 and IL-10 and the chemokines, CCL7, CCL11, CXCL12, and CXCL16, which contribute to the inflammatory process [55]. Furthermore, these cytokines are not only related to the establishment of a protumor microenvironment and organ-directed metastasis, but they also mediate disease progression by promoting tumor cell growth and proliferation, including HPV-induced malignancies [56]. In the present study, we found that *norank_o__Peptostreptococcales-Tissierellales* had a positive correlation with the p16 proportion. A previous report demonstrated that significantly different levels of *Peptostreptococcus* exist between oral dysplasia and OSCC [57]. Lissoni A et al. [58] also suggested that the main bacterial species that correlate with OSCC include *Peptostreptococcus.*

On a functional level, we predicted that viral carcinogenesis was more enriched in p16-positive TSCC, and correlation analysis showed that some signatures of bacterial families and species were associated with tumor lymphatic metastasis (*Prevotellaceae*) and muscle infiltration (*Pseudomonadaceae*). Therefore, for clinically p16-positive TSCC patients, paying attention to the changes of these two bacteria may have reference significance and be helpful for judging the malignancy grade, but this specificity and accuracy need further research. For *Prevotellaceae*, previous reports demonstrated that *Prevotellaceae* was significantly increased in OSCC [39], esophageal [59] and gastric cancer tissues [60]. Zhang et al. [61] showed that the abundance of *Prevotellaceae* was positively correlated with the severity of oral mucositis in patients with squamous cell carcinoma of the head and neck. Recently, Zhang et.al [62] performed a largest study to assess the association between the oral microbiome and oral HPV DNA, and found that oral HPV infection is associated with a higher abundance of *Prevotellaceae.* Mechanically, HPV-associated carcinogenesis is mediated through the influence of oncoprotein E6 and E7 expression on cell cycle regulatory pathways, which leads to p16 protein accumulation [63, 64]. Overexpression of p16 has been indicated as a marker for HPV-related cancer [17, 65] and p16 may be a clear potential marker in the detection of dysplasia in head and neck squamous mucosa [66]. Regarding *Pseudomonadaceae*, researchers revealed that *Pseudomonadaceae* was linked to a higher risk of developing OSCC [25] and pancreatic cancer [67]. In particular, women with high-risk HPV had significantly higher relative abundances of *Pseudomonadaceae* than those with low-risk-HPV or no HPV infection [68]. This indicates that these bacteria play important roles in HPV-associated cancer. Therefore, it is advised that additional research be done with a greater emphasis on these microorganisms in the p16-positive TSCC.

There are limitations to our study. First, although we started with an effort to collect 60 paraffin samples, the number of samples successfully sequenced for microbial sequencing was very small. This problem is also a thorny one that is currently faced with the use of paraffin samples for microbiome research. We used different optimization schemes, such as different primers and exploring suitable amplification conditions, etc., which can provide a reference for future research. Second, its retrospective design and consequent scope for missing information about risk factors such as smoking and alcohol, in addition to the absence of TNM stage, treatment outcome and survival state, prevented a more complete analysis. Previous studies have shown that smoking and alcohol consumption drive microbial changes particularly in the saliva [69, 70]. However, it is uncertain whether the microbiota status in TSCC tissue is similarly affected. Future investigations using large sample sizes are necessary to examine these potential risk factors and microbial alterations in TSCC.

In summary, we performed a small pilot study of microbiological studies of TSCC by paraffin tissue, and some challenges associated with DNA damage in FFPE tissues indeed exist. These results can be used as a reference for future workers who also use paraffin samples for research. Nevertheless, we have tentatively found some meaningful results. The p16 status is associated with microbiota diversity, this diversity was significantly increased in p16-positive patients compared with p16-negative patients. *Desulfobacteria, Limnochordia*, *Phycisphaerae, Anaerolineae*, *Saccharimonadia* and *Kapabacteria* had higher abundances among 16-positive participants. Moreover, functional prediction revealed that the increase in these bacteria may enhance viral carcinogenesis in p16-positive TSCC. These findings may provide insights into the relationship between p16 status and the microbial taxa in TSCC, and developing new drugs that target these bacteria may be a promising strategy for intervening in p16-positive TSCC progression.

### Electronic supplementary material

Below is the link to the electronic supplementary material.

Supplementary Material 1

Supplementary Material 2

Supplementary Material 3

Supplementary Material 4

## Data Availability

The datasets generated and analysed during the current study are available in The National Omics Data Encyclopedia(NODE) repository (Project ID: OEP003768, https://www.biosino.org/node/run/detail/OER376183).
