# Supplementary material for: Using formalin fixed paraffin embedded tissue to characterize the microbiota in p16-positive and p16-negative tongue squamous cell carcinoma: a pilot study
Source: BMC Oral Health. 2024 Feb 28;24:283. doi: 10.1186/s12903-024-04051-w (PMC10900712; doi:10.1186/s12903-024-04051-w)
Supplement: Supplementary file 1 — Supplementary Material 1 [file 12903_2024_4051_MOESM1_ESM.docx]

**Supplementary Table S1** **The clinic-pathological data of OSCC** **patients**

| **Primer** | **Patients** | **P16+** | **P16-** | **Age** | **Gender** | **Differentiation degree** | **Tumor lymphatic metastasis** | **Muscle infiltration** |
| --- | --- | --- | --- | --- | --- | --- | --- | --- |
| 515F/806R | Patients 1 |  | 🗸 | 44 | F | 2 | **-** | **-** |
|  | Patients 2 |  | 🗸 | 22 | F | 2 | **-** | **+** |
|  | Patients 3 |  | 🗸 | 69 | F | 2 | **-** | **+** |
|  | Patients 4 |  | 🗸 | 54 | F | 1 | **-** | **-** |
|  | Patients 5 |  | 🗸 | 67 | F | 1 | **-** | **-** |
|  | Patients 6 |  | 🗸 | 54 | M | 2 | **-** | **+** |
|  | Patients 7 | 🗸 |  | 56 | M | 2 | **-** | **+** |
|  | Patients 8 | 🗸 |  | 53 | F | 2 | **+** | **+** |
|  | Patients 9 | 🗸 |  | 47 | M | 2 | **-** | **+** |
|  | Patients 10 | 🗸 |  | 53 | M | 3 | **+** | **+** |
|  | Patients 11 | 🗸 |  | 43 | M | 1 | **-** | **-** |
|  | Patients 12 | 🗸 |  | 63 | F | 2 | **+** | **+** |
| 27F/338R | Patients 13 |  | 🗸 | 54 | F | 2 | **-** | **+** |
|  | Patients 14 |  | 🗸 | 45 | M | 1 | **-** | **+** |
|  | Patients 15 |  | 🗸 | 49 | M | 2 | **-** | **+** |
|  | Patients 16 |  | 🗸 | 55 | M | 2 | **+** | **+** |
|  | Patients 17 |  | 🗸 | 46 | F | 2 | **-** | **-** |
|  | Patients 18 |  | 🗸 | 60 | M | 3 | **+** | **+** |
|  | Patients 19 | 🗸 |  | 56 | M | 3 | **-** | **+** |
|  | Patients 20 | 🗸 |  | 43 | F | 2 | **-** | **+** |
|  | Patients 21 | 🗸 |  | 37 | M | 2 | **-** | **+** |
|  | Patients 22 | 🗸 |  | 54 | F | 1 | **-** | **-** |
|  | Patients 23 | 🗸 |  | 43 | M | 1 | **-** | **-** |
|  | Patients 24 | 🗸 |  | 67 | F | 1 | **-** | **-** |
|  | Patients 25 | 🗸 |  | 72 | M | 3 | **+** | **-** |
|  | Patients 26 | 🗸 |  | 45 | M | 1 | **+** | **-** |
|  | Patients 27 | 🗸 |  | 51 | M | 1 | **-** | **+** |

**Differentiation degree 1: well-differentiated TSCC; 2: moderately-differentiated TSCC; 3: poorly-differentiated TSCC.**

**Supplementary Table S2** **The effect of paraffin samples from different years on the success rate of amplification**

| **Primers** |  | **Paraffin embedding time** | | | **P-value** |
| --- | --- | --- | --- | --- | --- |
| **515F/806R** | **Grades of DNA quality** | **3 years** | **2 years** | **1 year** | P>0.05 |
|  | **A/B** | 8(29.6%) | 2(11.8%） | 2(12.5%) |  |
|  | **C** | 19 | 15 | 14 |  |
|  | **Total** | 27 | 17 | 16 |  |
| **27F/338R** | **A/B** | 7(25.9%) | 2(11.7%) | 6(37.5%) | P>0.05 |
|  | **C** | 20 | 15 | 10 |  |
|  | **Total** | 27 | 17 | 16 |  |

**Supplementary Table S3** **The effect of paraffin samples from different hospitals on the success rate of amplification**

| **Primers** | **Different hospitals** | **Second Affiliated Hospital of**  **Harbin Medical University** | **Stomatological Hospital of**  **China Medical University** | **P-value** |
| --- | --- | --- | --- | --- |
| **515F/806R** | **A/B** | 9 | 3 | P>0.05 |
|  | **C** | 23 | 25 |  |
|  | **Total** | 32 | 28 |  |
| **27F/338R** | **A/B** | 8 | 7 | P>0.05 |
|  | **C** | 24 | 21 |  |
|  | **Total** | 32 | 28 |  |
