# Supplementary material for: Using formalin fixed paraffin embedded tissue to characterize the microbiota in p16-positive and p16-negative tongue squamous cell carcinoma: a pilot study
Source: BMC Oral Health. 2024 Feb 28;24:283. doi: 10.1186/s12903-024-04051-w (PMC10900712; doi:10.1186/s12903-024-04051-w)
Supplement: Supplementary file 4 — Supplementary Material 4 [file 12903_2024_4051_MOESM4_ESM.docx]

**Additional file 1**

Supplementary tables S1 The clinic-pathological data of OSCC patients

Supplementary tables S2 The effect of paraffin samples from different years on the success rate of amplification

Supplementary tables S3 The effect of paraffin samples from different hospitals on the success rate of amplification

**Additional file 2**

Supplementary Table S4 Predicted the functional pathways by PICRUST(Sequence by primers 515F-806R)

Supplementary Table S5 Predicted the functional pathways by Tax4Fun (Sequence by primers 515F-806R)

**Additional file 3**

Supplementary Table S6 Predicted the functional pathways by PICRUST(Sequence by primers 27F-338R)

Supplementary Table S7 Predicted the functional pathways by Tax4Fun (Sequence by primers 27F-338R)
